# Glomerular abundance of complement proteins characterized by proteomic analysis of laser-captured microdissected glomeruli associates with progressive disease in IgA nephropathy

**DOI:** 10.1186/s12014-017-9165-x

**Published:** 2017-08-14

**Authors:** Teodora Ioana Flavia Paunas, Kenneth Finne, Sabine Leh, Hans-Peter Marti, Tom Eirik Mollnes, Frode Berven, Bjørn Egil Vikse

**Affiliations:** 1 Department of Medicine, Haugesund Hospital, Postbox 2170, 5504 Haugesund, Norway; 2 Department of Clinical Medicine, University of Bergen, Bergen, Norway; 3 Department of Pathology, Haukeland University Hospital, Bergen, Norway; 4 Department of Medicine, Haukeland University Hospital, Bergen, Norway; 5 Department of Immunology, Oslo University Hospital, Rikshospitalet, K. G. Jebsen Inflammation Research Center, University of Oslo, Oslo, Norway; 6 Research Laboratory Nordland Hospital, K. G. Jebsen Thrombosis Research and Expertise Center, University of Tromsø, Bodø, Norway; 7 Centre of Molecular Inflammation Research, Norwegian University of Science and Technology, Trondheim, Norway; 8 Department of Biomedicine, University of Bergen, Bergen, Norway

**Keywords:** IgA nephropathy, Complement, ESRD, Formalin-fixed paraffin embedded kidney biopsy tissue, Liquid chromatography–tandem mass spectrometry, Proteomic analyses

## Abstract

**Background:**

The clinical course of IgA nephropathy (IgAN) is variable and complement activation may predict prognosis. The present study investigated whether glomerular abundance of complement proteins associates with progression to end-stage renal disease (ESRD) in patients for whom prognosis could not be predicted based on clinical variables.

**Methods:**

Based on data from the Norwegian Kidney Biopsy Registry and the Norwegian Renal Registry, three groups were included: IgAN patients with (n = 9) or without (n = 16) progression to ESRD during 10 years, and controls (n = 15) with a normal kidney biopsy. IgAN patients had eGFR > 45 ml/min/1.73 m^2^ and non-nephrotic proteinuria at time of biopsy. Using stored formalin-fixed paraffin embedded kidney biopsy tissue, about 100 glomerular cross sections were microdissected for each patient. Samples were analyzed by liquid chromatography–tandem mass spectrometry and relative abundances of complement proteins were compared between groups.

**Results:**

Proteomic analyses quantified 2018 proteins, of which 28 proteins belong to the complement system. As compared to IgAN patients without progressive disease, glomeruli from patients with progressive IgAN had significantly higher abundance of components of the classical and the terminal complement pathways, and inhibitory factors such as Factor H and factor H related proteins. Abundance of complement proteins classified progressors from non-progressors with an area under ROC curve of 0.91 (*p* = 0.001). Clinical and morphological data were similar between the two patient groups and could not predict progressive IgAN.

**Conclusions:**

In conclusion, higher glomerular abundance of complement proteins was associated with a progressive clinical course in IgAN and are candidate biomarkers to predict prognosis.

**Electronic supplementary material:**

The online version of this article (doi:10.1186/s12014-017-9165-x) contains supplementary material, which is available to authorized users.

## Background

The clinical course of IgA nephropathy (IgAN) is highly variable and difficult to predict, some patients have a stable clinical course, while others progress to end-stage renal disease (ESRD). Several clinical and histological factors at time of diagnosis have been shown to indicate worse prognosis. These include low estimated glomerular filtration rate (eGFR), hypertension, proteinuria, mesangial hypercellularity, segmental glomerulosclerosis or adhesion, tubular atrophy and interstitial fibrosis [1–3]. There is however a large group with moderate risk in which individual prognostication based on these factors is difficult and there is a clear need for better prognostic markers in this group [4].

It has long been suggested that complement has an important role in the pathogenesis of IgAN as complement components C3, properdin and factor H have been commonly co-detected with IgA deposits in renal biopsy specimens [5–7]. Complement activation can occur through the classical, lectin or alternative pathways [8–11], that ultimately result in activation of the terminal complement pathway. Previous studies have shown that the lectin pathway [12, 13] and the alternative pathway [14] likely are involved in the pathophysiology of IgAN.

In the present study we investigated markers of progressive IgAN in patients with medium risk of progression based on eGFR and proteinuria. Patients were included in a case–control design comparing patients with progressive IgAN to non-progressive disease IgAN as well as to control patients. Glomerular cross sections were microdissected and glomerular protein abundances were compared between groups. Initial findings showed that complement related proteins seemed to be important and we thus compared abundances of these proteins in progressive versus non-progressive IgAN andnon-progressive IgAN versus healthy control patients and describe associations with clinical and morphological parameters. Lastly we investigate whether complement related proteins showed potential for prediction of progressive IgAN.

## Methods

The study was approved by the Regional Committee for Medical and Health Research Ethics.

### Registries used in the study

Data from the Norwegian Kidney Biopsy Registry were used for selection of patients. The registry has recorded clinical, biochemical and histopathological data at time of biopsy from nearly all patients who have undergone a non-neoplastic kidney biopsy in Norway since 1988. Serum creatinine, systolic blood pressure and urinary protein excretion at time of biopsy were used as reported to the registry. Creatinine was measured at the local hospital laboratories using kinetic Jaffe method until about 2005 when there was a swithch to the IDMS traceable enzymatic test, the switch was done at slightly different time points at different hospitals. In the present study, creatinine values measured before 2005 was recalculated based on a formula used by Hallan et al. to recalibrate creatinine to IDMS-traceble values [15]. We calculated eGFR based on the the CKD-EPI equation [16]. Urinary protein was quantified as g/24 h either from directly measured values, by calculation from reported urinary protein to creatinine ratio or if only reported by urinary dipstick a negative dipstick was set to 0 g/24 h, 1+ was set to 0.5 g/24 h, 2+ was set to 1.0 g/24 h and 3+ was set to 3.0 g/24 h [4]. By using the 11-digit national identity number, data from the Norwegian Kidney Biopsy Registry were linked with the Norwegian Renal Registry which has registed all cases with ESRD in Norway since 1980. At the time of linkage, data on ESRD were available until 2013.

### Study population

Based on data from the described registries, patients were selected for three subgroups. (1) Non-progressive IgAN, criteria: diagnosis of IgAN at kidney biopsy, eGFR > 45 ml/min/1.73 m^2^, urinary protein >1 g/24 h and no development of ESRD during a follow-up period of at least 10 years. (2) Progressive IgAN, criteria: diagnosis of IgAN at kidney biopsy, eGFR > 45 ml/min/1.73 m^2^, urinary protein <3.5 g/24 h and development of ESRD during the first 10 years after kidney biopsy. (3) Control patients, criteria: normal or minimal morphological changes in the kidney biopsy, eGFR > 60 ml/min/1.73 m^2^, urinary protein <0.5 g/24 h and no development of ESRD during a follow-up period of at least 10 years. All biopsies had been performed as part of a standard clinical work-up where glomerular disease was suspected. By review of medical journals, data on steroid treatment were retrieved for all IgAN patients and last available serum creatinine and urinary protein were also retrieved for patients who had not developed ESRD.

### Laser capture microdissection and sample preparation

The remaining part of the kidney biopsy core that was not used for diagnostic examination has been stored as formalin-fixed paraffin-embedded tissue and was used for the present study. Ten micrometer thick FFPE sections were deparaffinized, rehydrated and stained with haematoxylin–eosin. Glomeruli with global sclerosis, more than minimal segmental sclerosis, crescents or fibrinoid necrosis were excluded. Based on these criteria, eligible glomeruli were laser microdissected (PALM MicroBeam, Zeiss) and pressure catapulted into a tube cap (AdhesiveCap 500 clear, Zeiss). For each patient, we aimed to microdissect about 100 glomerular cross sections.

Microdissected FFPE glomeruli were suspended in 10 µL lysis solution and stored at −20 °C until peptide extraction. Protein extraction and trypsinization of microdissected glomeruli were performed as previously described [17].

### Liquid chromatography and tandem mass spectrometry

The samples were analyzed on a Q-Exactive HF (Thermo Scientific) connected to a Dionex Ultimate NCR-3500RS LC system. The MS instrument was equipped with an EASY-spray ion source (Thermo Scientific) and MS spectra were acquired as described in detail in the supplemental information documenting the detailed methods.

### Label free quantification

The raw data was analyzed with the Progenesis LC–MS software (version 4.0, Nonlinear Dynamics, UK) using default settings. Features were exported from Progenesis and imported into Proteome Discoverer (version 1.4, Thermo Scientific) for protein identification using the SwissProt human database (downloaded from UniProt August 2015, 20,197 sequences).

### Histology and immunohistochemistry

The biopsies were reclassified in a blinded manner by an experienced nephropathologist (SL) using the Oxford classification scoring system and M, E, S and T scores were assigned [3]. Immunohistochemistry was performed on 3 µm thick sections from FFPE tissue after antigen retrieval with proteinase digestion. The following antibodies were used: polycolonal rabbit anti-human C3c (Dako, Glostrup, Danmark; A0062), polyclonal rabbit-antihuman C1q (Dako, Glostrup, Danmark; A0136) and monoclonal mouse anti-human C5b-9, clone aE11 (Dako, Glostrup, Danmark; M0777). The aE11 antibody detects a neoepitope exposed in C9 after C9 is incorporated in the C5b-9 complex and is not present in native C9, thus specifically deting activation of the whole complement cascade [18]. Nearly all biopsies had been stained for C3c and C1q at time of diagnostic evaluation and these sections were used for evaluation. For C5b-9 staining, new sections were used. Glomerular positivity for complement factors was evaluated by semiquantitative scoring ranging from 0 to 3+.

### Statistics and bioinformatics

Clinical and morphological variables are described either as mean ± standard deviation or as percentages Tests of statistical significance were performed with *t*-tests or Chi-square statistics. Normalized protein abundances were compared between groups with *t*-tests and considered differently abundant if identified by at least two unique peptides and *p* value <0.05. Fold change is given for relative quantification of protein abundance between groups. Mean ± standard deviation is given where appropriate. Linear regression was performed to explore the relationship between complement proteins and clinical variables GFR, proteinuria and blood pressure.

A complement score was calculated for each IgAN patient by multiplying scores for all included complement proteins (score for each protein calculated as the protein abundance for the patient divided by mean protein abundance for all patients with IgAN; for proteins with fold change <1 in the comparison between IgAN with progression divided with IgAN without progression, the score was exponentiated by −1). The complement score was logarithmically transformed. Receiving operating characteristics (ROC) curves were used to evaluate the performance of the complement protein score and area under the curve (AUC) were calculated. Two complement scores were calculated, one including all significant complement proteins and one including only proteins of the MAC complex (complement factors C5, C6, C7, C8 and C9). ROC curves were also created for systolic blood pressure, complement component C7 and 1/eGFR for comparison.

## Results

Three groups were included and kidney biopsy tissue could be retrieved and enough glomeruli microdissected for 16 patients with non-progressive IgAN, 9 patients with progressive IgAN and 15 controls with normal biopsies. The clinical and morphological characteristics of the three groups are summarized in Table 1. There was no statistical significant difference in clinical characteristics between IgAN patients with progressive versus non-progressive disease. Oxford classification showed no difference in M, E or S score between patients with versus without progression, T score was however more often positive in patients with progressive disease (44 vs 0%) (*p* = 0.004).

**Table 1:** Clinical characteristics and MEST classification of included patients at time of biopsy

	Controls	IgAN without progression	IgAN with progression
N	15	16	9
Year of diagnosis	2000 ± 7.7	1996 ± 3.4	1998 ± 5.7
Proportion female	53.3%	12.5%	33.3%
Age (years)	32.0 ± 11.9	31.4 ± 13.4	31.2 ± 15.8
Serum creatinine (μmol/l)	79.3 ± 20.9	91.5 ± 21.5	105.8 ± 25.6*
Estimated glomerular filtration rate^a^ (ml/min/1.73 m^2^)	113.13 ± 18.5	114.19 ± 25.3	91.89 ± 26.3*
Systolic blood pressure (mmHg)	118.6 ± 14.6	127.2 ± 14.3	135.4 ± 25.8
Diastolic blood pressure (mmHg)	77.6 ± 8.4	78.9 ± 11.6	78.8 ± 13.1
Urinary protein (g/24 h)	0.16 ± 0.17	1.76 ± 1*	2.00 ± 1.98*
Body weight (kg)	74.7 ± 11.9	76.1 ± 8.0	75.4 ± 12.5
No of years of follow-up	12.3 ± 7.7	16.3 ± 3.4	
No of years from biopsy to ESRD			5.8 ± 2.5
Percentage with M-score of 1	Not applicable	31.3%	44.4%
Percentage with E-score of 1	Not applicable	31.3%	33.3%
Percentage with S-score of 1	Not applicable	50%	77.8%
Percentage with T-score of 1 or 2	Not applicable	0%	44.4%*

* *p* < 0.05 as compared to control. No variables were statistically significant between IgAN with versus without progression

^a^Estimated by CKD-EPI equation

### Glomerular proteome analysis

A total of 3274 proteins were identified, of which 2018 were identified with two or more unique peptides and could thus be used in quantitative analyses. Of these, 231 proteins had significant different abundance between progressive and non-progressive IgAN. The 25 most strongly significantly changed proteins in progressive versus non-progressive IgAN are listed in Table 2. Notably, 10 (40%) of these were complement proteins and we therefore chose to focus further studies towards complement proteins. In the list of all quantified proteins, 28 were complement proteins.

**Table 2:** List of the 25 proteins with the highest fold-change between IgAN with versus without progression, only significantly changed proteins included in list

UniProtKB accession	N unique peptides	Protein name	Fold change	*p* value
O00391	4	Sulfhydryl oxidase 1	3.33	0.004
P13671	16	Complement C6	2.95	0.0003
P10643	14	Complement C7	2.65	0.001
P08519	7	Apolipoprotein(a)	2.58	0.004
P11215	4	Integrin alpha-M	2.56	0.03
P00736	2	Complement C1r subcomponent	2.53	0.001
P01876	21	Ig alpha-1 chain C region	2.35	0.02
P01019	4	Angiotensinogen	2.26	0.03
P68032	2	Actin, alpha cardiac muscle 1	2.21	0.02
P0C0L5	5	Complement C4-B	2.20	0.01
Q9GIY3	2	HLA class II histocompatibility antigen, DRB1-14 beta chain	2.16	0.05
P22102	4	Trifunctional purine biosynthetic protein adenosine-3	2.07	0.002
P04114	32	Apolipoprotein B-100	2.01	0.04
P07358	14	Complement C8 beta chain	1.97	0.01
P07357	19	Complement C8 alpha chain	1.97	0.001
P01031	39	Complement C5	1.93	0.01
Q9HCU0	2	Endosialin	1.87	0.05
P04003	14	C4b-binding protein alpha chain	1.86	0.01
P31947	2	14-3-3 protein sigma	1.84	0.03
P07225	3	Vitamin K-dependent protein S	1.84	0.03
P36980	3	Complement factor H-related protein 2	1.84	0.003
O15143	7	Actin-related protein 2/3 complex subunit 1B	1.83	0.03
Q15063	10	Periostin	1.79	0.04
Q9BXR6	22	Complement factor H-related protein 5	1.79	0.01
Q9BY44	2	Eukaryotic translation initiation factor 2A	1.76	0.03

### Complement proteins in progressive versus non-progressive IgAN

In the comparison between progressive versus non-progressive IgAN, 18 complement proteins were significantly different, 17 proteins were more abundant and one (complement receptor 1, CR1) were less abundant (Table 3). Complement components that had significantly increased abundance were C1q, C1r, C1s, C3, C4-B, C5, C6, C7, C8 and C9. Complement regulators also had increased abundance, including clusterin, factor H, factor H-related proteins 2, 5 and C4b-binding protein alpha chain..

**Table 3:** Change in abundance for quantified complement and complement related proteins for progressive versus non-progressive IgAN and for non-progressive IgAN versus control

UniProt accession	N unique peptides	Protein name	Progressive versus non-progressive IgAN		Non-progressive IgAN versus control	
			Fold change	*p* value	Fold change	*p* value
*Complement system components*						
P02746	4	Complement C1q subcomponent subunit B	1.18	0.57	1.47	0.002
P02747	4	Complement C1q subcomponent subunit C	1.54	0.04	1.18	0.53
P00736	2	Complement C1r subcomponent	2.53	0.001	0.97	0.11
P09871	2	Complement C1s subcomponent	1.43	0.02	1.26	0.28
P00751	17	Complement factor B	1.06	0.68	1.01	0.74
P06681	2	Complement C2	1.36	0.13	1.07	0.57
P01024	105	Complement C3	1.46	0.01	1.93	0.0001
P0C0L4	44	Complement C4-A	1.31	0.45	1.25	0.01
P0C0L5	5	Complement C4-B	2.20	0.01	1.01	0.80
P01031	39	Complement C5	1.93	0.01	2.45	0.00002
P13671	16	Complement C6	2.95	0.0003	1.89	0.0005
P10643	14	Complement C7	2.65	0.001	3.76	0.00001
P07357	19	Complement C8 alpha chain	1.97	0.001	2.51	0.0003
P07358	14	Complement C8 beta chain	1.97	0.01	2.20	0.0003
P07360	10	Complement C8 gamma chain	1.72	0.0002	1.28	0.001
P02748	25	Complement C9	1.65	0.03	4.10	0.00004
*Complement system regulators*						
P13987	2	CD59 glycoprotein	1.26	0.14	1.29	0.07
P10909	14	Clusterin	1.68	0.0004	1.39	0.01
P08603	38	Complement factor H	1.34	0.02	1.58	0.0003
Q03591	9	Complement factor H-related protein 1	1.72	0.10	3.96	0.0002
P36980	3	Complement factor H-related protein 2	1.84	0.003	2.79	0.002
Q02985	4	Complement factor H-related protein 3	2.18	0.08	1.54	0.049
Q9BXR6	22	Complement factor H-related protein 5	1.79	0.01	2.67	0.0002
P17927	13	Complement receptor type 1	0.63	0.04	0.62	0.001
Q2VPA4	4	Complement component receptor 1-like protein	0.72	0.07	0.64	0.01
P04003	14	C4b-binding protein alpha chain	1.86	0.01	0.99	0.87
P04004	18	Vitronectin	1.16	0.18	1.75	0.001
P05155	4	Plasma protease C1 inhibitor	1.51	0.26	1.04	0.65

**Fig. 1:**
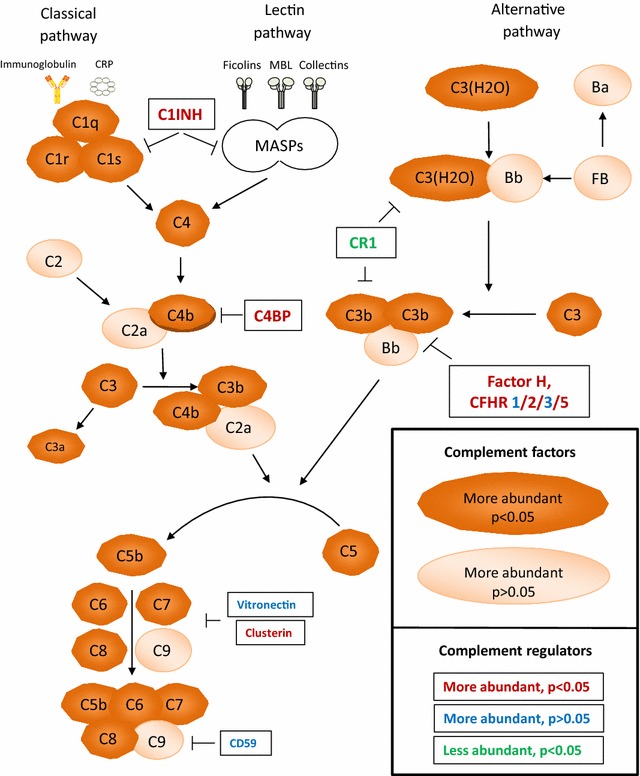
Illustration of the complement activation pathway with annotations for quantified glomerular proteins in progressive versus non-progressive IgAN patients. C1INH, plasma protease C1 inhibitor; C4BP, C4b-binding protein; CR1, Complement receptor type 1; FB, factor B; MASP, mannose-associated serine protease. All other abbreviations are names of the complement components or subcomponents

The proteins that were significantly changed in progressive versus non-progressive IgAN patients were mapped to the complement pathway (Fig. 1). Further analyses of the complement factor subcomponents of C2, C4 and C5 were unfortunately not possible due to few specific peptides. For C3 we did however identify 105 peptides, of which 65 peptides could be used for analysis of sub-components. Fold-change for specific C3 peptides were: C3 beta chain 1.30 (*p* = 0.002), C3c alpha1 chain 1.22 (*p* = 0.3), C3c alpha-2 chain 1.30 (*p* = 0.002) and C3dg 2.16 (*p* = 0.000005) (Additional file 1: Table S1).

### Complement proteins in non-progressive IgAN versus controls

In the comparison between patients with non-progressive IgAN and controls, 19 proteins were significantly differently abundant, 17 were more abundant and two were less abundant (Table 3), a similar pattern to that observed for the comparison between progressive and non-progressive IgAN.

### Immunohistochemistry

**Fig. 2:**
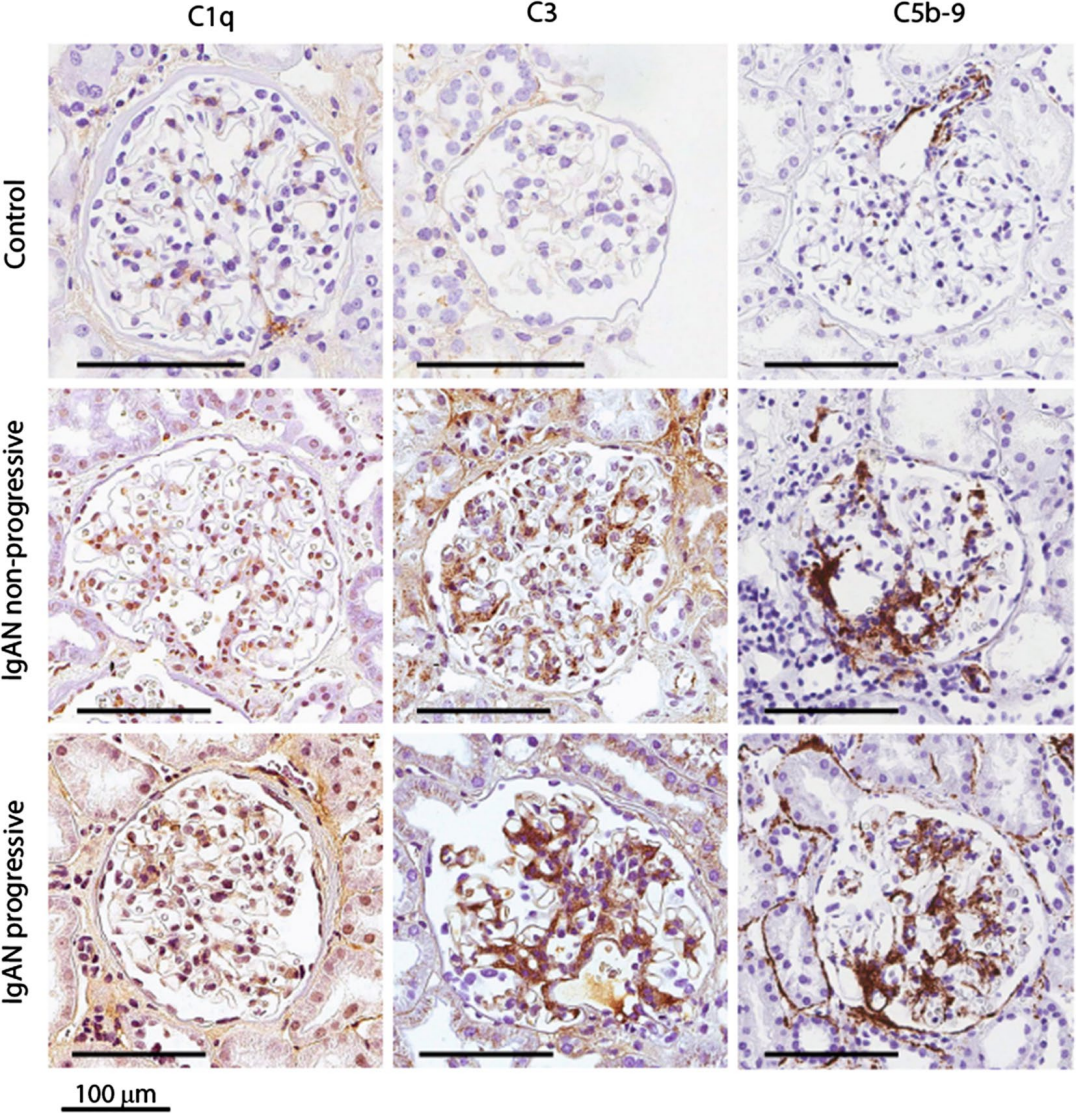
Representative immunohistochemistry staining images for complement factors C1q, C3 and membrane attack complex (C5b-9) for a representative control, a representative patient with non-progressive IgAN and a representative patient with progressive IgAN

Representative pictures illustrating immunohistochemistry staining for the three groups are shown in Fig. 2. Scores for glomerular staining for C1q was similar between the groups (0.57 ± 0.78 in controls, 0.43 ± 0.64 in non-progressive IgAN and 0.38 ± 0.52 in progressive IgAN). Scores for C3 staining were different between groups (0.14 ± 0.38, 0.71 ± 0.61 and 1.38 ± 0.91, respectively; *p* for trend 0.005, *p* = 0.055 for comparison progressive versus non-progressive IgAN). Scores for C5b-9 were 0 for all control patients but similar between non-progressive and progressive IgAN patients (0.64 ± 1.02 vs 0.71 ± 1.11).

### Prediction of progressive versus non-progressive IgAN

**Fig. 3:**
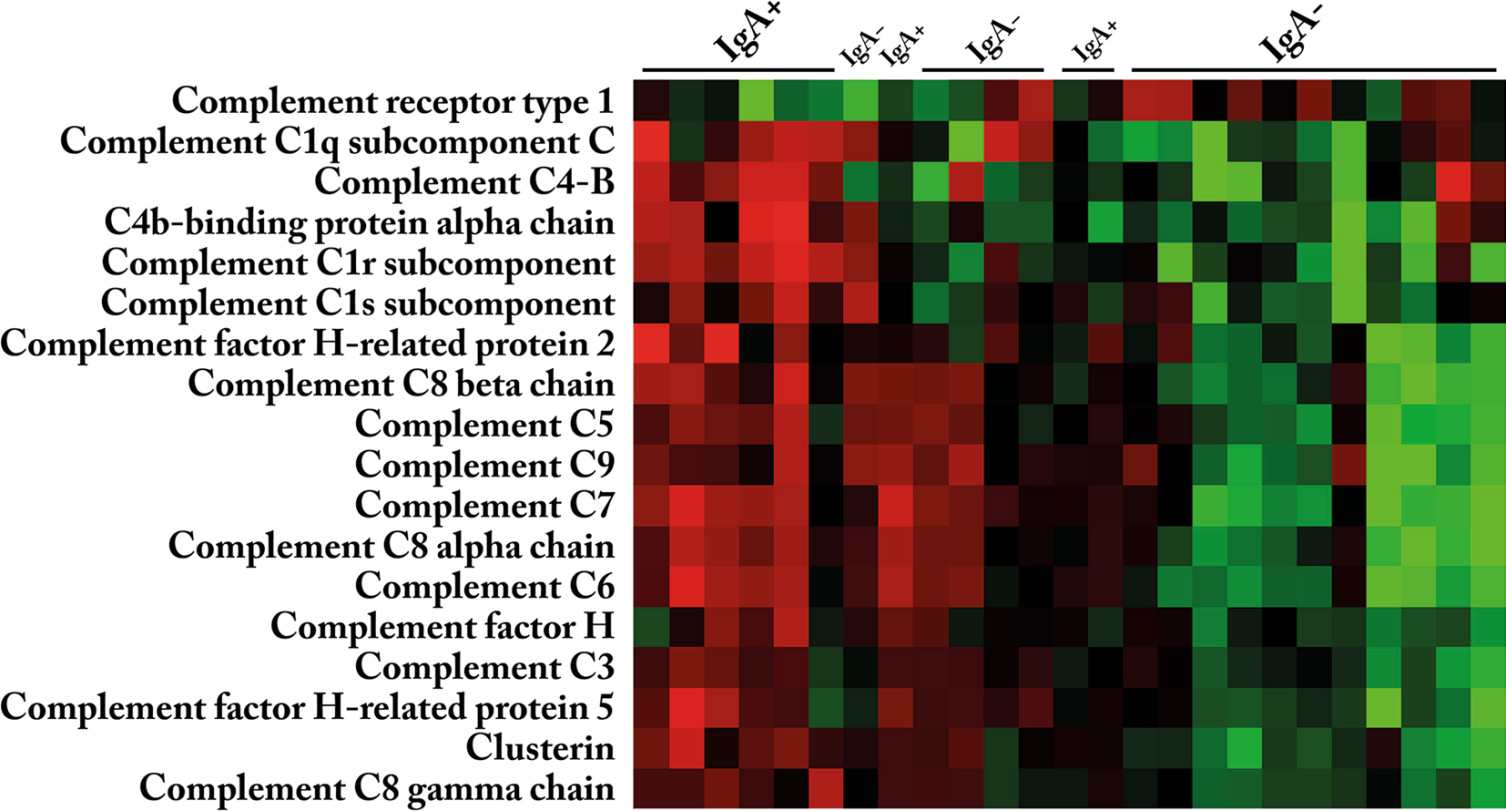
Hierarchical clustering for proteins significantly different between progressive (IgA+) versus non-progressive IgAN (IgA−)

As shown above, glomerular protein abundance of complement proteins were higher in patients with IgAN with progressive disease as compared to IgAN with non-progressive disease. We further analysed whether glomerular abundance of these proteins could classify IgAN patients as progressive versus non-progressive. Unsupervised hierarchical clustering including only the significantly abundant complement related proteins of Table 3 separated most patients with progressive and non-progressive disease (Fig. 3).

**Fig. 4:**
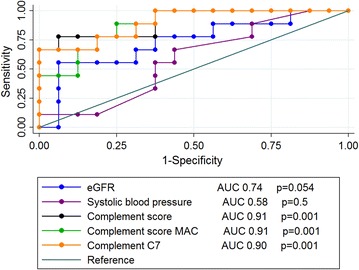
ROC curves for complement scores, systolic blood pressure and eGFR

A complement score was calculated for each patients based on abundance of complement related proteins (for details, see “Methods” section). Patients with progressive IgAN had significantly higher scores than patients with non-progressive IgAN, and controls had lower scores than non-progressive IgAN. We further tested whether these scores could be used to classify patients with progressive versus non-progressive IgAN. In ROC analyses, AUC values were 0.91 (*p* = 0.001) for a complement score using all significant proteins, 0. 91 (*p* = 0.001) for the complement score including complement components C5, C6, C7, C8 and C9 and 0.90 (*p* = 0.001) when only including protein abundance of complement factor C7, the rate limiting factor of the terminal pathway (Fig. 4). In comparison, AUC value for the clinical variables systolic blood pressure was 0.580 (*p* = 0.5) and for the variable 1/eGFR it was 0.74 (*p* = 0.054). Other clinical or morphological variables could neither be used to classify progressive from non-progressive IgAN.

### Associations between complement proteins and clinical variables

For patients with IgAN, linear associations between the complement proteins and clinical variables were investigated. These analyses showed that C1r, C1s, C5, C6, C8, C9 and clusterin had higher abundance with lower eGFR (Table 4). There were no significant associations with urinary protein, but there were increased abundances of the C1r, C1s, C4, C5, C8, C9, factor H, factor H-related protein 3 and C4b binding protein alpha with increasing systolic blood pressure.

**Table 4:** Linear associations between complement related proteins significantly altered in Table 3 in the comparison progressive versus non-progressive IgAN and clinical variables at time of biopsy

	eGFR		Urinary protein		Systolic blood pressure	
	B*	*p* value	B*	*p* value	B*	*p* value
*Complement system components*						
Complement C1q subcomponent subunit C	−	0.3	+	0.2	+	0.5
Complement C1r subcomponent	−	0.003	+	0.5	+	0.02
Complement C1s subcomponent	−	0.005	+	0.7	+	0.003
Complement C3	−	0.3	−	0.5	+	0.2
Complement C4-B	−	0.2	+	0.9	+	0.05
Complement C5	−	0.04	−	0.3	+	0.02
Complement C6	−	0.03	−	0.3	+	0.09
Complement C7	−	0.06	−	0.5	+	0.2
Complement C8 alpha chain	−	0.03	−	0.4	+	0.05
Complement C8 beta chain	−	0.02	−	0.2	+	0.004
Complement C8 gamma chain	−	0.1	+	0.6	+	0.4
Complement C9	−	0.01	−	0.24	+	0.01
*Complement system regulators*						
Clusterin	−	0.02	−	0.3	+	0.1
Complement factor H	−	0.1	−	0.5	+	0.02
Complement factor H-related protein 1	−	0. 4	−	0.8	+	0.1
Complement factor H-related protein 2	+	0.9	−	0.9	+	0.5
Complement factor H-related protein 5	−	0.3	−	0.5	+	0.5
Complement receptor type 1	+	0.07	+	0.9	−	0.4
C4b-binding protein alpha chain	−	0.06	−	0.4	+	<0.001

Only IgAN patients

* Direction of association is shown, + means higher intensity with higher value for clinical marker and − means lower intensity with higher value for clinical marker

### Associations between complement proteins and MEST score

Distribution of MEST scores are shown in Table 1. Complement levels were compared between IgAN patients with positive as compared to negative scores for the 4 different MEST characteristics (Table 5). Positive M score was associated with higher abundance of complement proteins C5, C6, C7, C8, and clusterin, and lower abundance of complement receptor type 1. Positive E score was associated with higher abundance of C5, C7, C9 and complement factor H-related protein 5. Positive S score was associated with higher abundance of C1r and C1s. Positive T score was associated with higher abundance of C1q, C1r, C1s, C4, C5, C6, C7, C8 clusterin, complement factor H and C4b-binding protein, and lower abundance of complement receptor type 1.

**Table 5:** Glomerular complement protein abundance in IgAN patients according to MEST score; only proteins significant in Table 3 are included

	M1 versus M0		E1 versus E0		S1 versus S0		T1/2 versus T0	
	Fold change	*p* value	Fold change	*p* value	Fold change	*p* value	Fold change	*p* value
*Complement system components*								
Complement C1q subcomponent subunit C	0.9	0.88	1.1	0.57	1.1	0.71	2.7	0.02
Complement C1r subcomponent	0.8	0.81	1.5	0.37	1.6	0.03	2.7	0.02
Complement C1s subcomponent	0.9	0.76	1.7	0.05	1.5	0.04	2.0	0.01
Complement C3	1.5	0.05	1.5	0.06	0.9	0.86	1.5	0.15
Complement C4-B	1.1	0.75	0.8	0.21	1.3	0.65	2.6	0.01
Complement C5	2.5	0.03	2.3	0.02	1.1	0.57	2.3	0.04
Complement component C6	2.1	0.04	1.7	0.07	0.9	0.69	2.7	0.02
Complement component C7	2.2	0.02	2.4	0.03	1.1	0.55	3.3	0.03
Complement component C8 alpha chain	2.3	0.02	2.2	0.05	1.0	0.64	2.4	0.04
Complement component C8 beta chain	2.0	0.06	2.0	0.05	0.9	0.98	1.8	0.07
Complement component C8 gamma chain	1.7	0.002	1.4	0.05	1.2	0.47	1.6	0.01
Complement component C9	2.3	0.06	2.2	0.04	0.9	0.67	1.9	0.07
*Complement system regulators*								
Clusterin	1.8	0.003	1.4	0.14	1.2	0.28	1.9	0.01
Complement factor H	1.4	0.09	1.5	0.07	1.3	0.33	1.7	0.01
Complement factor H-related protein 1	2.3	0.07	2.2	0.16	1.2	0.85	2.4	0.10
Complement factor H-related protein 2	1.7	0.11	1.8	0.07	1.2	0.90	1.5	0.20
Complement factor H-related protein 5	1.6	0.09	1.4	0.04	0.9	0.94	1.6	0.19
Complement receptor type 1	0.5	0.003	0.6	0.10	0.8	0.30	0.4	0.001
C4b-binding protein alpha chain	0.9	0.67	1.2	0.60	1.2	0.16	2.6	0.001

## Discussion

In the current study we have shown that patients with progressive IgAN had higher glomerular abundance of complement proteins as compared to patients with non-progressive IgAN. Interestingly, both ordinary complement components and most of the complement inhibitors showed higher abundance, indicating compensatory mechanisms taking place during activation. IgAN patients selected for the present study had medium risk of progression and prognosis could not be predicted based on accepted risk factors such as eGFR, proteinuria, blood pressure or the Oxford classification. Glomerular abundance of all significant complement proteins, in particular those of the terminal pathway, did however show predictive performance with area under the ROC curve of about 0.9. Similar findings for complement proteins were made when comparing non-progressive IgAN patients to controls, indicating a dose–response relationship.

In the present study we were able to quantify 28 complement proteins. We found increased abundance of proteins related to the classical and terminal pathway. Members of the terminal pathway (complement factors C5–C9) that constitute the MAC, showed the strongest increase in progressive versus non-progressive IgAN as well as in non-progressive IgAN versus controls. Previous studies have shown increased glomerular MAC deposition [19] and increased urinary MAC levels [20] in IgA nepropathy. The prognostic importance has however not been shown before. Local expression of terminal pathway components in renal cells has not been described [21] indicating that our finding are suggestive of complement activation and not just local synthesis. In Fig. 2 we show mesangial localization of the membrane attack complex with an antibody against a neoepitope in C9 that only stain positive for the assembled complex, indicating activation of the complex and not just deposition of the native component.

In our study, components of the classical pathway C1q, C1r and C1s, were significantly increased in patients with progressive IgAN as compared with non progressive IgAN, suggesting the involvement of the classical pathway in the progression of the diseases. In our study, we could not detect MASP (mannose binding lectin associated serine proteases), MBL (mannose binding lectin) or ficolins and we could thus not find evidence for activation of the lectin pathway. We thus suggest that the increased abundance of complement component C4 in progressive IgAN may argue for contribution of the classical pathway in IgAN patients with progressive disease.

Furthermore, complement C3 mesangial deposition was also significantly increased in progressive IgAN. The alternative pathway is suggested to be activated in IgAN as complement C3 mesangial deposition is present in >90% of patients and Immunglobulin A has been shown to activate the alternative pathway in vitro [7, 22]. As C3 is present both by activation of the classical and the lectin pathtay by the amplification loop, it is not possible to know with certainty whether or not the alternative pathway was activated primarily in IgGAN. Interestingly, analyses of the subcomponents of C3 showed stronger increase of C3dg than the other peptides in progressive IgAN. C3dg is an inactive product of degraded C3b and our findings thus indicate increased opsonization by C3b in patients with progressive IgAN. Similar findings of accumulation of C3dg was recently also shown for C3 glomerulopathy [23]. Other regulators of the complement system, such as factor H, which is one of the most important regulators of C3 and the alternative pathway, were also mostly significantly increased in progressive IgAN. These findings suggest that compensatory mechanisms are active in IgAN in order to control the increased complement activation. One inhibitor of the complement system, complement receptor 1 (CR1) that acts by inactivating C3b and is localized on the podocytes [24] was however present in lower abundance in progressive IgAN. Previous studies have shown reduced CR1 in injured podocytes from patients with different types of glomerulopathies [25] and one study also showed reduced CR1 expression in lupus nephritis [26]. The decrease in CR1 may contribute to a disturbed balance with increased activation and reduced inhibition, enhancing the detrimental effects of complement activation in IgAN. The exact mechanisms for complement activation and regulation in IgAN cannot howver be mapped by the present study, but the clear evidence of its prognostic role points to a need for further studies.

In the selection of IgAN patients for the present study, we aimed to include patients with medium risk of progression and a progressive versus non-progressive disease course. The rationale for the selection criteria based on eGFR and proteinuria was to select patients in whom prediction of prognosis was difficult based on traditional risk factors and indeed, prognosis could not be predicted based on classical risk factors. Initially, we planned to include only patients with proteinuria of 1–3.5 g/24 h, but due to a limited number of patients with these characteristics, we chose to add 3 patients with proteinuria less than 1 g/24 h who progressed to ESRD and 1 patient with proteinuria above 3.5 g/24 h who did not progress to ESRD. In our opinion this approach yielded two groups with progressive versus non-progressive disease for whom prediction of prognosis was very difficult, in strong line with the rationale described above.

Complement score, either based on abundance of significant complement proteins, and in particular components of the membrane attack complex, could however predict prognosis with area under ROC curve of about 0.9. Unsupervised hierarchical clustering also showed the same, confirming and strengthening these findings. Two important reservations should however be made. First, the predictive capacity could not be reproduced with immunohistochemistry staining for C5b-9, and staining for C3 was only moderately increased in patients with progressive IgAN, the direct clinical significance should therefore be interpreted with caution. Second, we investigated the predictive ability of the complement scores in the same cohort in which we demonstrated the importance and not in a separate cohort. Our results therefore need confirmation in a new cohort. A previous study also showed prognostic importance of C4d staining, this staining was not tested in our study [27].

The most important strengths of the present study are the relevant study population with IgAN in whom the prognosis was difficult to predict, microdissection and analysis of the relevant glomerular tissue, the large number of quantified proteins and the dose–response relationships that were seen for progressive IgAN versus non-progressive IgAN versus controls.

## Conclusions

In conclusion, the present study has shown increased abundance of complement factors and inhibitors in progressive IgAN as compared to non-progressive IgAN. Increased abundance of proteins of the terminal complement pathway argue for complement-mediated damage in progressive IgAN. One inhibitor of the complement system, CR1, had lower abundance in progressive IgAN and may represent a mechanism that reduces complement inhibitory control in IgAN.

## Additional file

**Additional file 1: Table** **S1.** Quantified sub-components of complement factor C3 in progressive vs non-progressive IgAN.
