# Supplementary material for: Glomerular abundance of complement proteins characterized by proteomic analysis of laser-captured microdissected glomeruli associates with progressive disease in IgA nephropathy
Source: Clin Proteomics. 2017 Aug 14;14:30. doi: 10.1186/s12014-017-9165-x (PMC5557313; doi:10.1186/s12014-017-9165-x)
Supplement: Supplementary file 1 — Additional file 1: Table S1. Quantified sub-components of complement factor C3 in progressive vs non-progressive IgAN. [file 12014_2017_9165_MOESM1_ESM.docx]

Additional file 1: Table S1. Quantified sub-components of complement factor C3 in progressive vs non-progressive IgAN.

|  | N peptides | Sequence coverage (%) | Fold change | p-value |
| --- | --- | --- | --- | --- |
| C3 beta chain | 26 | 54.6 | 1.30 | 0.002 |
| C3c alpha fragment 1 | 7 | 33.0 | 1.22 | 0.3 |
| C3c alpha fragment 2 | 10 | 36.7 | 1.30 | 0.002 |
| C3dg | 20 | 66.8 | 2.16 | 0.000005 |
